# Peanut Can Be Used as a Reference Allergen for Hazard Characterization in Food Allergen Risk Management: A Rapid Evidence Assessment and Meta-Analysis

**DOI:** 10.1016/j.jaip.2021.08.008

**Published:** 2022-01

**Authors:** Paul J. Turner, Nandinee Patel, Barbara K. Ballmer-Weber, Joe L. Baumert, W. Marty Blom, Simon Brooke-Taylor, Helen Brough, Dianne E. Campbell, Hongbing Chen, R. Sharon Chinthrajah, René W.R. Crevel, Anthony E.J. Dubois, Motohiro Ebisawa, Arnon Elizur, Jennifer D. Gerdts, M. Hazel Gowland, Geert F. Houben, Jonathan O.B. Hourihane, André C. Knulst, Sébastien La Vieille, María Cristina López, E.N. Clare Mills, Gustavo A. Polenta, Natasha Purington, Maria Said, Hugh A. Sampson, Sabine Schnadt, Eva Södergren, Stephen L. Taylor, Benjamin C. Remington

**Affiliations:** a National Heart & Lung Institute, Imperial College London, London, United Kingdom; b Allergy Unit, Department of Dermatology, University of Zürich, Zürich, Switzerland; c Clinic for Dermatology and Allergology, Kantonsspital St Gallen, St Gallen, Switzerland; d Food Allergy Research and Resource Program, Department of Food Science and Technology, University of Nebraska, Lincoln, Neb; e Netherlands Organisation for Applied Scientific Research TNO, Utrecht, the Netherlands; f Brooke-Taylor & Co Pty Ltd, Milawa, Vic, Australia; g Children's Allergy Service, Evelina Children's Hospital, Guy’s and St. Thomas’ NHS Foundation Hospital, London, United Kingdom; h Department of Paediatric Allergy, King’s College London, London, United Kingdom; i Department of Allergy and Immunology, the Children’s Hospital at Westmead, Westmead, NSW, Australia; j DBV Technologies, Montrouge, France; k State Key Laboratory of Food Science and Technology, Sino-German Joint Research Institute, Nanchang University, Nanchang, China; l Sean N. Parker Center for Allergy and Asthma Research, Stanford University School of Medicine, Stanford, Calif; m René Crevel Consulting Limited, Bedford, United Kingdom; n GRIAC Research Institute, University of Groningen, University Medical Center Groningen, Groningen, the Netherlands; o Clinical Research Center for Allergy and Rheumatology, National Hospital Organization, Sagamihara National Hospital, Sagamihara, Japan; p Institute of Allergy, Immunology and Pediatric Pulmonology, Yitzhak Shamir Medical Center, Zerifin, Israel; q Department of Pediatrics, Sackler Faculty of Medicine, Tel Aviv University, Tel Aviv, Israel; r Food Allergy Canada, Toronto, ON, Canada; s Allergy Action, St Albans, United Kingdom; t Department of Paediatrics, Royal College of Surgeons in Ireland and Children’s Health Ireland Temple St Hospital, Dublin, Ireland; u Department Dermatology/Allergology and Center for Translational Immunology, University Medical Centre Utrecht, Utrecht University, Utrecht, the Netherlands; v Food Directorate, Health Canada, Ottawa, ON, Canada; w Food Engineering Department, San Martín National University, Buenos Aires, Argentina; x Division of Infection, Immunity and Respiratory Medicine, Manchester Institute of Biotechnology, University of Manchester, Manchester, United Kingdom; y Instituto Nacional de Tecnología Agropecuaria (INTA), Instituto Tecnología de Alimentos, Buenos Aires, Argentina; z Department of Medicine, Quantitative Sciences Unit, Stanford University School of Medicine, Stanford, Calif; aa Allergy & Anaphylaxis Australia, Castle Hill, NSW, Australia; bb Division of Pediatric Allergy and Immunology, Icahn School of Medicine at Mount Sinai, New York, NY; cc Deutscher Allergie- und Asthmabund (DAAB), Mönchengladbach, Germany; dd Livsmedelsverket (Swedish Food Agency), Uppsala, Sweden; ee ThermoFisher Scientific, Uppsala, Sweden; ff Remington Consulting Group BV, Utrecht, the Netherlands

**Keywords:** Anaphylaxis, Eliciting dose, Food allergy, Precautionary allergen labeling, Reference dose, Threshold, CI, Confidence interval, DBPCFC, Double-blind, placebo-controlled food challenge, ED, Eliciting dose, ED_01_, Amount of allergen expected to cause objective symptoms in 1% of the population with that allergy, ED_05_, Amount of allergen expected to cause objective symptoms in 5% of the population with that allergy, EIA, Exercise-induced anaphylaxis, FAO, Food and Agriculture Organization, FC, Food challenge, PAL, Precautionary allergen labeling, PFAS, Pollen food allergy syndrome, WHO, World Health Organization

## Abstract

Regional and national legislation mandates the disclosure of “priority” allergens when present as an ingredient in foods, but this does not extend to the unintended presence of allergens due to shared production facilities. This has resulted in a proliferation of precautionary allergen (“may contain”) labels (PAL) that are frequently ignored by food-allergic consumers. Attempts have been made to improve allergen risk management to better inform the use of PAL, but a lack of consensus has led to variety of regulatory approaches and nonuniformity in the use of PAL by food businesses. One potential solution would be to establish internationally agreed “reference doses,” below which no PAL would be needed. However, if reference doses are to be used to inform the need for PAL, then it is essential to characterize the hazard associated with these low-level exposures. For peanut, there are now published data relating to over 3000 double-blind, placebo-controlled challenges in allergic individuals, but a similar level of evidence is lacking for other priority allergens. We present the results of a rapid evidence assessment and meta-analysis for the risk of anaphylaxis to a low-level allergen exposure for priority allergens. On the basis of this analysis, we propose that peanut can and should be considered an exemplar allergen for the hazard characterization at a low-level allergen exposure.

In most jurisdictions, regional and national legislation mandates the disclosure of “priority” allergens when present as an ingredient in foods.^1^ However, this does not extend to the unintended presence of allergens due to the use of shared production facilities. Attempts to alert consumers of this have contributed to a proliferation of precautionary allergen (“may contain”) labeling (PAL) that poses considerable difficulties to food-allergic consumers, in part because of a lack of transparency in terms of what PAL actually means.^2^ Attempts have been made by industry and regulators to improve allergen risk management to better inform the use of PAL, but to date this has not resulted in a consistent approach; indeed, this is now leading to discordance as some national regulators take different approaches to others.^1^^,^^3^ This lack of harmonious approach means that food businesses currently use PAL in different ways.^2^^,^^3^

Currently, there is no global consensus on what levels of allergen exposure cause harm to food-allergic consumers; this is needed to develop a regulatory approach. One strategy has been to establish internationally agreed “reference doses,” below which no PAL would be needed.^3^, ^4^, ^5^, ^6^ This would provide a regulatory framework for more appropriate and evidence-based use of PAL. Diverse stakeholders including patient representative groups consider that this would result in better informed food choices and thus better protect consumers with food allergy—many of whom currently ignore PAL due to their widespread (and arguably, over-) use and uncertainty in interpretation.^1^^,^^2^^,^^7^ There is now a significant evidence base to inform population thresholds for eliciting dose (ED), the dose of allergen predicted to provoke reactions in a defined proportion of the food-allergic population. For example, the amount of allergen expected to cause objective symptoms in 5% of the population with that allergy (ED_05_) is a dose predicted to provoke an objective allergic reaction in 5% of the at-risk allergic population.^6^ Such data can and have been used to inform the need for PAL, albeit on a voluntary basis.^4^^,^^5^ However, until now, the main consideration has been the proportion of allergic individuals who will have objective symptoms at these levels of exposure, and not necessarily a consideration of how “severe” such symptoms may be. Thus, there is also a need to better characterize the hazard^8^^,^^9^ at a given dose—the relationship between a level of allergen exposure (dose) and the nature/severity of any subsequent adverse health outcome—because this relationship is perceived to be of critical importance by allergic consumers and remains the focus of clinical, scientific, and regulatory debate.

Under current European legislation, food may be considered “unsafe” if injurious to health, for example, due to the “particular health sensitivities of a specific category of consumers” such as those with food allergies. However, what constitutes “injurious to health” is not precisely defined; indeed, interpretation of the law indicates that provided a food product is labeled in accordance with legal requirements (ie, including priority allergens where appropriate), food is safe, unless it is specifically marketed for people with those health sensitivities.^10^ In Canada, food is also considered to be unsafe if it contains undeclared food allergens, whether as an ingredient or unintended presence due to shared production facilities^11^; however, the requirement for allergen declaration “does not apply to a food allergen or gluten that is present in a prepackaged product as a result of cross-contamination.”^12^ The Food Allergen Labeling and Consumer Protection Act (2004) in the USA more explicitly enshrines the concept of an “allergic response that causes a risk to human health,” which implies that some reactions might not pose such a risk.^13^ By definition, therefore, there is a hierarchy of risks faced by people with food allergy, some of which might not be considered to be a risk to human health.^14^ Fatal food anaphylaxis is the most extreme harm that can occur, but fortunately, it is a very rare event, occurring at less than 1 per 100,000 person-years in food-allergic individuals (Figure 1).^15^ Investigating fatal reactions is extremely difficult, as it is usually impossible to determine the amount of allergen that has been consumed or the presence of other factors that might have contributed to the fatal outcome (although to date, there are no reports of fatal reactions to levels of exposure not exceeding the ED_05_ for any allergenic food).^20^ Although fatal reactions can theoretically occur to any allergen, the vast majority of fatal reactions reported in the literature are due to peanut, tree nuts, seafood, and cow’s milk.^21^ Furthermore, such severe reactions are usually due to the consumption of nonprepacked foods (foods from restaurants, bakeries, takeaway or fast-food outlets, etc.)^20^^,^^22^; these foods are unlikely to have had a PAL statement in the same way as is the case with prepacked foods. The rarity of fatal reactions and their limited relevance in the context of managing unintended allergen presence makes fatal reactions an inappropriate basis for characterizing the hazard posed by such presence. Furthermore, allergic consumers want more than “just” protection from fatal reactions, given that moderate allergic reactions can be very unpleasant, even if not themselves life-threatening.

**Figure 1:**
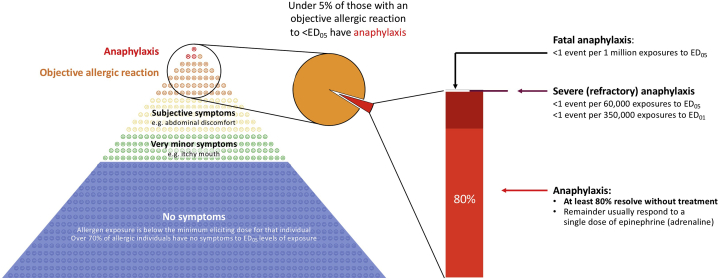
Hierarchy of risks faced by people susceptible to IgE-mediated food allergy. Estimates refer to occurrence of allergic symptoms at ED_05_ levels of exposure in food-allergic individuals.^15^, ^16^, ^17^, ^18^, ^19^*ED*_*05*_, Amount of allergen expected to cause objective symptoms in 5% of the population with that allergy.

If fatal reactions are not appropriate as an outcome for hazard characterization, arguably it is more important to protect the consumer from severe reactions at a population level. However, the assignment of severity for food-induced allergic reactions is inconsistent in the literature, and each method has its limitations.^23^^,^^24^ There is no universally accepted system for scoring the severity of food-allergic reactions. However, most food-allergic consumers and clinicians would consider reactions involving airway/breathing and/or cardiovascular compromise as severe, and there is an international consensus that such symptoms constitute “anaphylaxis” (despite there being multiple definitions for anaphylaxis in the literature).^24^ Notwithstanding, even nonfatal anaphylaxis is not a single entity in terms of severity (Figure 1). Published data indicate that at least 80% of anaphylaxis reactions are not treated with epinephrine/adrenaline (contrary to international guidelines), but resolve spontaneously.^16^^,^^17^ Although nontreatment must not to be condoned, it does demonstrate the spectrum of severity for anaphylaxis, from mild reactions that spontaneously resolve to more severe reactions refractory to initial treatment (occurring in 3.4% [95% confidence interval (CI): 1.9%-5.9%] of treated reactions).^18^

If reference doses (such as ED_05_) are to be used to inform the need for PAL or other risk management options, then it is essential to characterize the hazard associated with these low-level exposures. Assessing the risk of anaphylaxis to a low-dose allergen exposure would therefore seem to be appropriate in terms of this hazard characterization. For example, after an exposure to an ED_05_ amount of peanut (an amount which would, by definition, not cause an objective allergic reaction in 95% of peanut-allergic individuals), one would expect 2.3 episodes of anaphylaxis per 1000 exposures in the peanut-allergic population.^19^ At least 80% of these are mild reactions (which, in reality, resolve spontaneously when allergic individuals choose not to follow medical advice and treat), whereas 97% of the remainder would respond to first-line treatment (with epinephrine/adrenaline).^18^ In those reporting anaphylaxis to any level of exposure for a food allergen, the risk of fatal outcome is estimated to be <1:10,000^15^; it is likely that this rate would be even lower after an ED_05_ level of exposure. Therefore, the expected rate of fatal reaction to an ED_05_ exposure in an allergic individual can be estimated to be <1 per million (Figure 1). There are currently no reports in the literature of fatal reactions to this level of exposure, for any allergenic food.

For peanut, there are now published data relating to over 3000 double-blind, placebo-controlled challenges in allergic individuals to inform a reference dose and characterize the hazard associated with an ED_05_ exposure.^19^ However, for other priority allergens currently defined by the Food and Agriculture Organization/World Health Organization (FAO/WHO) Codex Alimentarius (cereals containing gluten; crustacea, egg, fish, soybean, milk, tree nuts), this level of evidence (both quantity and quality; eg, from double-blind challenges) is lacking, which results in more uncertainty in the estimate of the rate of anaphylaxis to low-level allergen exposures to these allergens. If peanut can be considered a “worst-case” allergen in terms of hazard and risk characterization at low levels of exposure, then this would greatly facilitate attempts to define reference doses and introduce a consistent regulatory framework for the use of PAL acceptable at an international level.

In this rostrum, we propose that “anaphylaxis” can be used as a “marker” for hazard characterization, that is, severity. We present the results of a rapid evidence assessment^25^ and meta-analysis evaluating the proportion of reactions to a low-dose allergen exposure that result in anaphylaxis for other priority allergens (see this article’s Online Repository at www.jaci-inpractice.org for methodology), to assess the evidence base and evaluate the uncertainty in the evidence (by comparing the 95% CIs for the rate of anaphylaxis at low-level exposures for peanut and other priority allergens). We conclude that despite the lower level of evidence—both quantity and quality (eg, not just from a double-blind, placebo-controlled food challenge [DBPCFC])—for allergens other than peanut, there are no data to suggest that other priority allergens cause “more severe” reactions at an ED_05_ level of exposure. On this basis, we therefore propose that peanut can and should be considered an exemplar allergen for hazard characterization at a low-level allergen exposure.

## Peanut

**Figure 2:**
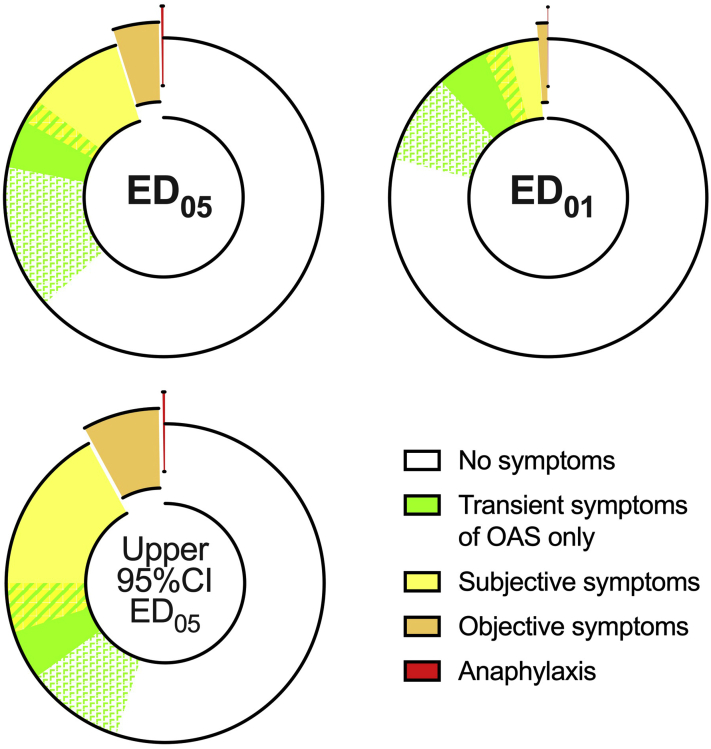
Proportion of peanut-allergic individuals expected to have subjective or objective symptoms after an exposure to an ED_05_ or ED_01_ amount of peanut. Data from Table I. *CI*, Confidence interval; *ED*_*01*_, amount of allergen expected to cause objective symptoms in 1% of the population with that allergy; *ED*_*05*_, amount of allergen expected to cause objective symptoms in 5% of the population with that allergy; *OAS*, oral allergy symptoms.

Patel et al^19^ recently published a systematic review of over 3000 DBPCFCs to peanut. This analysis found that approximately 4.5% (95% CI: 1.9%-10.1%) of individuals who reacted to ≤5 mg of peanut protein and 4.2% (95% CI: 0.7%-22.3%) of individuals who reacted to ≤1 mg with objective symptoms experienced anaphylaxis (exposures that approximate to the upper limit of the 95% CI for the amount of allergen expected to cause objective symptoms in 1% of the population with that allergy [ED_01_] and ED_05_ for peanut, respectively).^6^ A further 3 reports were identified with respect to subjective symptoms experienced after a low-dose peanut exposure at food challenge (FC). In the Peanut Allergen Threshold Study, 378 unselected peanut-allergic children underwent an open, single-dose challenge to 1.5 mg of peanut protein; 67 (17.7%; 95% CI: 14%-22%) developed subjective symptoms.^26^ Two further series provide dose-distribution curves for any (subjective + objective) symptoms at a DBPCFC to peanut.^27^^,^^28^ The latter also reported that at cumulative doses of 0.33 to 3.33 mg of peanut protein, around 5% to 10% of peanut-allergic individuals will experience mild transient oral allergy symptoms.^28^ At an ED_05_ exposure, around one-third of peanut-allergic individuals experience subjective symptoms (the vast majority of a mild and transient nature); 5% will have objective symptoms (equivalent to 50 peanut-allergic individuals per 1000), and only 4.5% of those (equivalent to 2.3 per 1000) are predicted to develop anaphylaxis (Table I and Figure 2).

**Table I:** Proportion of peanut-allergic individuals who would be expected to have symptoms after an exposure to an ED_01_ or ED_05_ amount of peanut

Peanut	1 mg of protein (≈upper 95% CI for cumulative ED_01_)	2.1 mg of protein (=discrete ED_05_)	7.1 mg of protein (=upper 95% CI for cumulative ED_05_)
Any symptom (subjective or objective)	14%^27^ to 23%^28^	20%^27^ to 35%^28^	35%^27^ to 45%^28^
Subjective symptoms	13%^27^ to 22%^28^	15% to 30%^26^, ^27^, ^28^	27%^27^ to 37%^28^
OAS only	5% to 10%^28^	5% to 10%^28^	5% to 10%^28^
Any objective symptom	1%	5%	8%^6^
Anaphylaxis rate:			
• In those reacting to this dose with objective symptoms	4.2%^19^ (95% CI: 0.7%-22.3%)	4.5%^19^ (95% CI: 1.9%-10.1%)	
• Overall, in the peanut-allergic population	0.04%^19^ (95% CI: 0.01%-0.22%)	0.23%^19^ (95% CI: 0.1%-0.5%)	

*CI*, Confidence interval; *ED*_*01*_, amount of allergen expected to cause objective symptoms in 1% of the population with that allergy; *ED*_*05*_, amount of allergen expected to cause objective symptoms in 5% of the population with that allergy; *OAS*, oral allergy symptoms.

The cumulative ED_01_ and ED_05_ for peanut is 0.7 (95% CI: 0.5-1.3) mg of protein and 3.9 (95% CI: 2.8-7.1) mg of protein, respectively; the discrete ED_05_ is 2.1 (95% CI: 1.2-4.6) mg of protein.^6^ Estimates of the occurrence of different symptoms are based on the literature.^26^, ^27^, ^28^

## Tree Nuts

Three studies were identified in which nut-allergic patients underwent a formal FC to a range of tree nuts. In the Pronuts study, a multicenter European study, 122 children (median age, 5.5 years) underwent multiple open FCs to peanut, tree nut, or sesame to assess coexistent allergy.^29^ A total of 689 FCs to tree nuts were performed, of which 191 (28%) were positive. Of 35 individuals who reacted to the first challenge dose (≤30 mg of protein), only 2 (5.7%) had anaphylaxis (Table II). Purington et al^30^ undertook a retrospective analysis of 410 individuals (median, 9 years; range, 1-52 years) who underwent DBPCFCs at 7 sites in the USA, which included 512 positive challenges to tree nuts. Severe symptoms were seen at all dosing levels, with no evidence to suggest that rates of anaphylaxis were greater for tree nuts at any level of exposure compared with peanut (Table III). After peanut, cashew and pecan were associated with the highest rates of anaphylaxis.

**Table II:** Positive food challenges in the Pronuts study^29^

	Total positive food challenges (n = 238)	No. reacting to ≤30 mg of protein	Anaphylaxis to ≤30 mg of protein	Symptoms
Almond	6/69 (9%)	0/6		
Brazil	7/100 (7%)	0/7		
Cashew	36/83 (43%)	10/36	0/10	
Hazelnut	30/70 (43%)	6/30	0/6	
Macadamia	16/100 (16%)	3/16	1/3	Laryngeal + lower respiratory symptoms
Pecan	26/92 (28%)	5/26	0/5	
Pistachio	34/94 (36%)	4/34	0/4	
Walnut	36/81 (44%)	7/36	1/7	Pruritic rash, local angioedema, stridor
Peanut	37/66 (56%)	8/37	0/8

**Table III:** Proportion of positive food challenges (to any dose) associated with symptoms consistent with anaphylaxis in Purington et al^30^

	No. of positive food challenges	Airway obstruction	Wheezing	Cardiovascular symptoms
Almond	30/44 (68%)	0%	0%	0%
Cashew	151/312 (48%)	1.3%	6.0%	0.7%
Hazelnut	68/95 (72%)	0%	2.9%	0%
Pecan	88/165 (53%)	2.3%	9.1%	0%
Pistachio	60/93 (65%)	1.7%	3.3%	0%
Walnut	121/195 (62%)	0%	2.5%	0%
Peanut	347/795 (44%)	1.2%	8.1%	0.3%

Further data relating to pecan and cashew can be found in the NutCracker study.^31^ In this study, 83 patients (median age, 8.7 years; range, 3-24 years) were prospectively evaluated for allergy to walnut, pecan, cashew, pistachio, hazelnut, and almond.^31^ Although patients did not undergo a challenge to peanut, rates of lower respiratory symptoms and/or the need for rescue epinephrine due to reactions across the entire FC dosing range were not greater than those reported in the literature for peanut.

**Figure E1:**
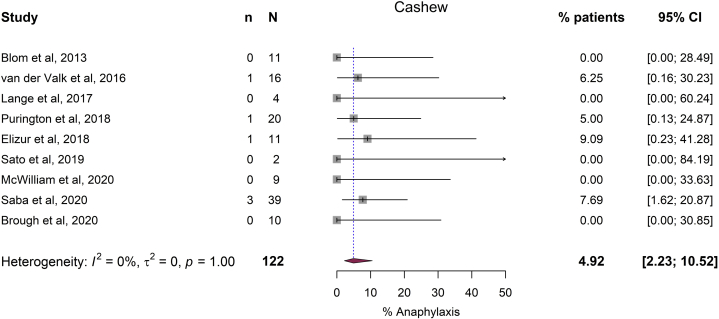


**Figure E2:**
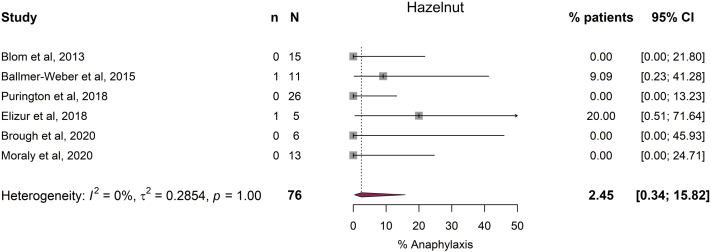


**Figure E3:**
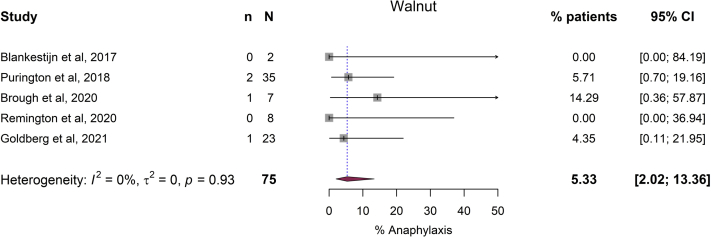


Finally, we assessed the rate of anaphylaxis to very low (≤upper 95th CI for the ED_05_)^6^ levels of allergen consumption at FC to cashew^27^^,^^29^, ^30^, ^31^, ^32^, ^33^, ^34^, ^35^, ^36^ (Table E1, available in this article’s Online Repository at www.jaci-inpractice.org), hazelnut^27^, ^28^, ^29^, ^30^, ^31^^,^^37^, ^38^, ^39^, ^40^ (Table E2, available in this article’s Online Repository at www.jaci-inpractice.org), and walnut^29^^,^^30^^,^^41^, ^42^, ^43^ (Table E3, available in this article’s Online Repository at www.jaci-inpractice.org) reported in the literature, and undertook a meta-analysis (Figures E1-E3, available in this article’s Online Repository at www.jaci-inpractice.org). These data are summarized in Table IV. Hazelnut was associated with a higher rate of subjective symptoms at lower doses compared with peanut—something not unexpected, because it is commonly implicated as a cause of pollen food allergy syndrome (PFAS) due to Bet v 1 cross-reactivity with birch pollen.^39^ This is also consistent with data published by Masthoff et al^38^ that after a low-dose exposure to hazelnut (≤10 mg of protein), subjective symptoms are almost twice as common in adults (in whom PFAS is more common) than in children. Overall, we found no evidence to suggest that tree nut-allergic individuals are more likely to experience anaphylaxis to low levels of exposure to a tree nut, compared with peanut.

**Table IV:** Proportion of individuals allergic to cashew, hazelnut, and walnut who would be expected to have symptoms after oral consumption of an ED_05_ amount

	Cashew	Hazelnut	Walnut
Discrete ED_05_	0.8 mg	3.5 mg	0.8 mg
Cumulative ED_05_	1.6 mg [95% CI: 0.4-9.4 mg]	4.7 mg [95% CI: 1.7-15.7 mg]	1.2 mg [95% CI: 0.1-13.0 mg]

	0.8 mg	9.4 mg	3.5 mg	15.7 mg	0.8 mg	13.0 mg
Any symptom (subjective or objective)	8%^27^ to >46%^32^	32%^27^	31%^27^ to 50%^28^	73%^28^ to 76%^27^	approx. 8%^27^	approx. 60%^27^
Subjective symptoms	3%^27^ to 46%^32^	20%^27^ to 66%^36^	26%^27^ to 48%^28^	67%^27^ to 70%^28^	Not known	approx. 46%^27^
OAS only	11%^32^	Not known	20%-30%^28^			Not known
Any objective symptom (based on ED_05_ definition)	5%	12%^6^	5%	9%^6^	5%	14%^6^
Estimated rate of anaphylaxis:						
• In those reacting to ≤ED_05_ exposure	4.9% (95% CI: 2.2%-10.5%)		2.5% (95% CI: 0.3%-15.8%)		5.3% (95% CI: 2.0%-13.0%)	
• Overall, in individuals with that specific food allergy	0.25% (95% CI: 0.11%-0.53%)		0.12% (95% CI: 0.02%-0.79%)		(95% CI: 0.10%-0.67%)	
Estimates based on:	597 FCs (318 DBPCFCs, 279 open FCs)		434 FCs (391 DBPCFCs, 43 open FCs)		350 FCs (194 DBPCFCs, 156 open FCs)	

*CI*, Confidence interval; *DBPCFC*, double-blind, placebo-controlled food challenge; *ED*_*05*_, amount of allergen expected to cause objective symptoms in 5% of the population with that allergy; *FC*, food challenge; *OAS*, oral allergy symptoms.

Estimates of the occurrence of different symptoms are based on the literature.

## Sesame

**Figure E4:**
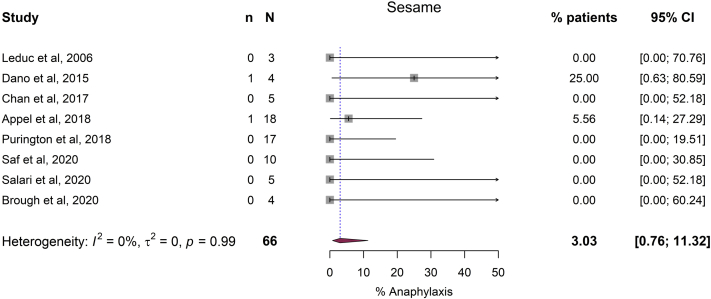


Sesame is already a priority allergen in the European Union, Canada, Australia, and New Zealand; the FASTER Act was recently passed in the USA, adding sesame to the list of priority allergens that must be declared when present as an ingredient in foods. Nine published studies were identified for sesame FCs (Table E4, available in this article’s Online Repository at www.jaci-inpractice.org), representing 271 positive FCs.^29^^,^^30^^,^^44^, ^45^, ^46^, ^47^, ^48^, ^49^, ^50^ Although some objective reactions were reported to low levels of exposure, only 2 (0.7%) anaphylaxis reactions were reported to <60 mg level exposures (equivalent to upper 95% CI for ED_05_ for sesame).^6^ At meta-analysis, this rate was equivalent to that for peanut, with a rate of anaphylaxis to ED_05_ levels of exposure of 3.0% (95% CI: 0.8%-11%) for sesame (Figure E4, available in this article’s Online Repository at www.jaci-inpractice.org).

## Cow’s Milk

**Figure E5:**
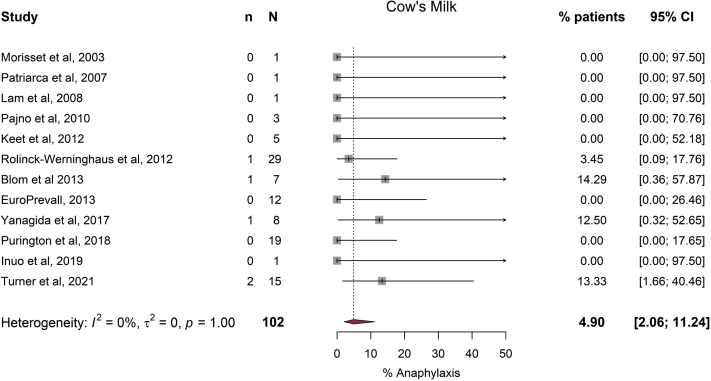


Seventeen studies were identified representing 1045 positive FCs (98% in children) (Table E5, available in this article’s Online Repository at www.jaci-inpractice.org).^27^^,^^30^^,^^51^, ^52^, ^53^, ^54^, ^55^, ^56^, ^57^, ^58^, ^59^, ^60^, ^61^, ^62^, ^63^, ^64^, ^65^ At meta-analysis, the estimated rate of anaphylaxis in those individuals reacting with objective symptoms to ED_05_ levels of exposure was 4.9% (95% CI: 2.1%-11%) (Figure E5, available in this article’s Online Repository at www.jaci-inpractice.org). Two studies also reported the occurrence of subjective symptoms to low-level exposures. Blom et al^27^ estimated that 13% to 20% of individuals with an allergy to cow’s milk will develop subjective symptoms to ED_05_ exposures (2.4-6.6 mg of cow’s milk protein). Turner et al^51^ reported a single-dose challenge study in which 50 of 172 milk-allergic individuals (29%) developed any symptoms to 0.5 mg of cow’s milk protein; at least 19% developed transient subjective symptoms, consistent with the estimate of Blom et al.

Although cow’s milk allergy is one of the most common food allergies in early childhood, the majority of children tend to outgrow it. This may explain why there is a perception that cow’s milk allergy is less “serious” than other food allergies.^66^^,^^67^ In reality, there are different phenotypes and children with persisting cow’s milk allergy may be more at risk of severe reactions: indeed, cow’s milk is the single most common cause of fatal anaphylaxis in children in the United Kingdom^68^ and a common cause of fatal and near-fatal reactions elsewhere.^21^

## Hen’s Egg

**Figure E6:**
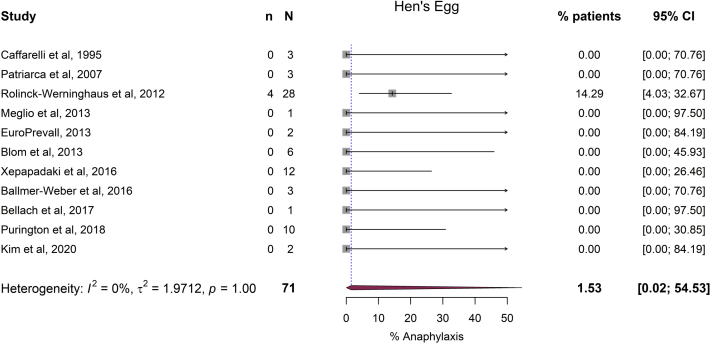


Twenty studies were identified (Table E6, available in this article’s Online Repository at www.jaci-inpractice.org), representing 1180 positive FCs, the vast majority of which (at least 95%) were in children.^27^^,^^30^^,^^48^^,^^53^^,^^58^^,^^62^^,^^63^^,^^69^, ^70^, ^71^, ^72^, ^73^, ^74^, ^75^, ^76^, ^77^, ^78^, ^79^, ^80^, ^81^, ^82^ At meta-analysis, the estimated rate of anaphylaxis in those individuals reacting with objective symptoms to ED_05_ levels of exposure was 1.5% (95% CI: 0.02%-55%) (Figure E6, available in this article’s Online Repository at www.jaci-inpractice.org). One study (Blom et al^27^) also provided an estimate of the occurrence of any symptoms to ED_05_ levels of exposure of 9% to 14% (which includes both subjective and objective symptoms). Data suggest that egg tends to cause less anaphylaxis (lower respiratory symptoms) and more gastrointestinal symptoms compared with other allergens.^83^ There are only 2 fatalities to egg reported in the literature,^21^^,^^68^^,^^84^ despite egg being one of the most common food allergens in preschool children.

## Wheat

**Figure E7:**
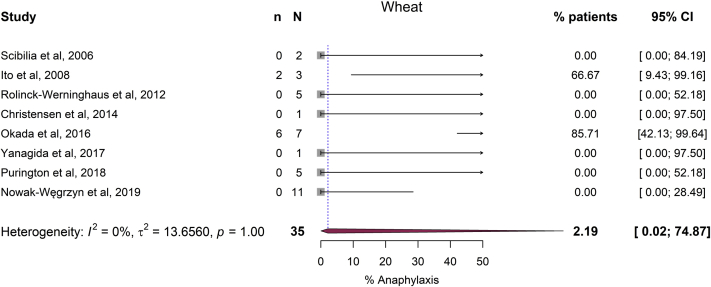


IgE-mediated wheat allergy is a relatively uncommon food allergy with a prevalence of under 0.5% in both children and adults^21^; celiac disease and non–IgE-mediated wheat allergy are more common. However, near-fatal and fatal anaphylaxis have been reported.^21^^,^^85^^,^^86^ Furthermore, wheat anaphylaxis may be more associated with anaphylactic shock (involving cardiovascular compromise) than other food allergens.^85^ Ten studies were identified, representing 348 positive FCs (at least 90% in children) (Table E7, available in this article’s Online Repository at www.jaci-inpractice.org).^30^^,^^53^^,^^58^^,^^86^, ^87^, ^88^, ^89^, ^90^, ^91^, ^92^ At meta-analysis, the rate of anaphylaxis in those individuals reacting with objective symptoms to ED_05_ levels of exposure was estimated to be 2.2% (95% CI: 0.02%-75%) (Figure E7, available in this article’s Online Repository at www.jaci-inpractice.org). Wheat is also the most common food allergen implicated in food-dependent, exercise-induced anaphylaxis (EIA).^85^ The available literature suggests that exposure levels causing wheat-dependent EIA in the presence of a relevant cofactor are typically in excess of those triggering reactions in conventional IgE-mediated wheat allergy.^93^^,^^94^

## Fish and Shellfish

Threshold data relating to fish and shellfish are limited, in part because of the multiple different species of seafood globally and limited published threshold data across these foods. Moreover, fish and shellfish are reported to have much higher reaction thresholds compared with other food allergens. Despite this, seafood is an emerging and important cause of anaphylaxis, including near-fatal and fatal anaphylaxis globally.^21^ Data from EuroPrevall indicate that around one-third of individuals allergic to seafood would experience subjective symptoms to an ED_05_ level of exposure of cod or prawn/shrimp.^28^ We identified 6 studies in the literature, 3 with respect to finned fish (typically cod)^28^^,^^95^^,^^96^ (Table E8, available in this article’s Online Repository at www.jaci-inpractice.org) and 3 evaluating thresholds to prawn/shrimp (Table E9, available in this article’s Online Repository at www.jaci-inpractice.org).^28^^,^^97^^,^^98^ With the paucity of data, no meta-analysis could be performed. The lack of data also results in wide estimated CIs for estimated ED_05_. Although anaphylaxis has been reported to ED_05_ levels of exposure, there is no evidence that this occurs more frequently than with peanut; however, the underlying evidence base is far more limited for this food group.

## Soybean

The inclusion of soya as a priority allergen in Codex is under review, with a recent recommendation from an FAO/WHO Expert Committee for its removal as a global priority allergen on the basis of a low level of prevalence and low frequency as a cause of anaphylaxis.^99^ For soybean, 5 studies were identified in the literature (Table E10, available in this article’s Online Repository at www.jaci-inpractice.org).^27^^,^^58^^,^^100^, ^101^, ^102^ Consistent with data suggesting that soybean is an uncommon cause of anaphylaxis globally,^21^ no cases of anaphylaxis to low (<200 mg of protein) levels of exposure were identified.

## Reproducibility of Thresholds and Likelihood of Anaphylaxis

Patel et al^19^ analyzed data from 534 individuals who underwent at least 2 peanut-DBPCFCs over time, to assess the reproducibility of thresholds and recurrence of anaphylaxis in peanut-allergic individuals. Although the intraindividual variability in ED varied by up to 3-log, in the majority 71.2% (95% CI: 56.2%-82.6%) of individuals, this was limited to a half-log change—equivalent to a single dosing interval when using a PRACTALL-style semilogarithmic dosing regimen.^103^ There was a similar degree of variability in the reproducibility of the dose at which participants experienced anaphylaxis; reassuringly, although some peanut-allergic individuals tolerated an ED_05_ exposure on one occasion but then reacted on another, no such subject developed anaphylaxis in this cohort. There are very limited data on the reproducibility of clinical thresholds for allergens other than peanut. Limited data (n ≈ 20) have been published for egg^69^ and wheat,^87^ with no evidence of increased variability in threshold compared with peanut (although for egg, the study was in children exposed to “baked” egg on a regular basis).

**Figure 3:**
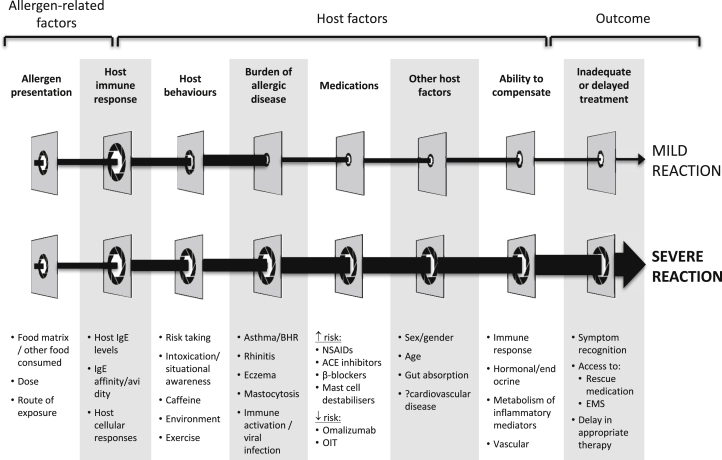
Factors that can modulate severity of allergic reactions. Reprinted with permission from Dubois et al.^14^*ACE*, Angiotensin converting enzyme; *BHR*, bronchial hyperresponsiveness; *EMS*, emergency medical services; *NSAID*, nonsteroidal anti-inflammatory drug; *OIT*, oral immunotherapy.

Multiple factors can impact on the severity of food-induced allergic reactions, as outlined in Figure 3. These include cofactors or “augmentation” factors such as exercise, stress, medication, sleep deprivation, and alcohol that appear to alter both the threshold at which individuals experience symptoms and the severity of symptoms at any given level of exposure.^14^^,^^104^ Importantly, these cofactors are not universal and inconsistently experienced by individuals; in many, if not most individuals, the most well-described factors (exercise, menstruation, alcohol) seem not to impact significantly on reaction severity.^104^ In a retrospective survey of almost 500 adults with food allergy, only a small proportion used medication that could influence severity, and under 10% reported exercise or alcohol as a relevant factor in reactions due to inadvertent exposure.^105^ The same study group recently published a prospective evaluation of accidental reactions in 157 patients over a 1-year period. Although 74% of reactions had at least 1 potential cofactor, there was no relationship between the presence of a cofactor and reaction severity.^106^

The TRACE peanut study evaluated the impact of significant exercise and sleep deprivation on peanut-induced allergic reactions in 100 peanut-allergic adults, using a randomized study design.^107^ On the basis of statistical modeling (rather than raw data), the authors reported a significant impact of both cofactors on reducing clinical thresholds by 45%. However, this decrease—around a ½-log, equivalent to a single dosing interval when using a PRACTALL-style semilogarithmic dosing regimen—is well within the intraindividual variation in reaction threshold reported by Patel et al.^19^ Indeed, in the TRACE study, the factor with the largest impact in threshold variability was the clinical center at which participants were evaluated. Furthermore, exercise was only identified as a significant factor in 1 of the 2 clinical centers.^107^^,^^108^ To date, no data relating to reaction severity from the TRACE study have been published. Therefore, although there can be an impact of cofactors on thresholds and severity in some individuals, this does not appear to be any greater than the inherent shift in both clinical thresholds and risk of anaphylaxis identified in the wider food-allergic population, nor does it appear that such effects are predictable. Consumers with food-dependent EIA (predominantly to wheat and possibly seafood) may be an exception: such individuals appear to be tolerant to the allergen in the absence of the relevant cofactor. However, at least for wheat-dependent EIA, EDs for clinical reaction are typically 2 to 3 log greater than ED_05_ levels of exposure.^93^^,^^94^

## Summary and Conclusions

There is a consensus that “zero risk” is not realistic or achievable with respect to food allergen risk management.^109^ An evidence-based approach to the use of PAL should improve both allergen risk communication *to* food-allergic consumers and their understanding and application *of* this information (different outcomes, but of equal importance). However, the reference doses used to inform the use of PAL must be guided by the residual “tolerable risk” and supported by current methods of allergen detection and risk management.

In this rapid evidence assessment and meta-analysis, we found no evidence to suggest that other priority allergens can result in a higher rate of anaphylaxis at low doses of allergen exposure (at around ED_05_ levels of exposure, which would be expected to cause objective symptoms in 5% of individuals allergic to that specific allergen), compared with peanut (Table V). Furthermore, we did not identify any cases of anaphylaxis at ≤ED_05_ levels that were refractory to treatment (where administered). Indeed, for many of the reports included in this analysis, a significant proportion of anaphylaxis reactions were not treated with epinephrine/adrenaline (reflecting both local variations in interpretation of anaphylaxis criteria and management of reactions by clinicians). At these low levels of exposure, the probability of anaphylaxis would be expected to be ≤0.25%. At least 80% of these episodes would resolve without treatment, whereas >97% of the remainder would respond to first-line treatment (with epinephrine/adrenaline). The risk of a fatal reaction to an ED_05_ exposure is estimated to be <1 per million; to date, there are no reports in the literature of fatal reactions to this level of exposure, for any allergenic food.

**Table V:** Summary table for the rate of anaphylaxis to ED_05_ levels of exposure in allergic individuals

Allergen	Evidence base (no. of FCs included in dataset)	Discrete ED_05_ (mg protein) [95% CI]	Upper limit of the 95% CI for cumulative ED_05_ (mg protein)	Expected rate of symptoms to a level of allergen exposure ≤upper 95% CI for the cumulative ED_05_		Expected rate of anaphylaxis to an allergen exposure ≤ upper 95% CI for the cumulative ED_05_, as a proportion of	
				Any symptoms (%)	Objective symptoms (%)	Individuals reacting to ED_05_ exposure with objective symptoms	All individuals allergic to this food
Peanut	3151 DBPCFCs	2.1 [1.2-4.6]	7.1	35-45	8	4.5% (95% CI: 1.9%-10%)	2.3 per 1000 (95% CI: 1.0-5.1 per 1000)
Cashew	323 DBPCFCs 421 open FCs	0.8 [0.2-5.0]	9.4	32	12	4.9% (95% CI: 2.2%-10.5%)	2.5 per 1000 (95% CI: 1.1-5.3 per 1000)
Hazelnut	391 DBPCFCs 43 open FCs	3.5 [1.3-12.1]	15.7	approx. 75	9	2.5% (95% CI: 0.3%-15.8%)	1.2 per 1000 (95% CI: 0.2-7.9 per 1000)
Walnut	194 DBPCFCs 156 open FCs	0.8 [0.1-8.9]	13.0	approx. 60	14	5.3% (95% CI: 2.0%-13%)	2.7 per 1000 (95% CI: 1.0-6.7 per 1000)
Sesame	59 DBPCFCs 214 open FCs	2.7 [0.4-34]	58	Not reported	20	3.0% (95% CI: 0.8%-11%)	1.5 per 1000 (95% CI: 0.4-5.7 per 1000)
Cow’s Milk	728 DBPCFCs 317 other FCs	2.4 [1.3-5.0]	6.6	20	9	4.9% (95% CI: 2.1%-11%)	2.5 per 1000 (95% CI: 1.1-5.5 per 1000)
Egg	637 DBPCFCs 543 other FCs	2.3 [1.2-4.7]	5.3	14	9	1.5% (95% CI: 0.02%-55%)	0.8 per 1000 (95% CI: 0-27 per 1000)
Wheat	123 DBPCFCs 23 open FCs	6.1 [2.6-15.6]	25	Not reported	11	2.2% (95% CI: 0.02%-75%)	1.1 per 1000 (95% CI: 0-38 per 1000)
Fish	59 DBPCFCs	12.1 [4.5-44]	102	58	25	Insufficient data for meta-analysis	
Shrimp	12 DBPCFCs 46 open FCs	280 [69-880]	1850	57	19	Insufficient data for meta-analysis	
Soya	89 DBPCFCs 51 open FCs	10.0 [2.2-55]	76	Not reported	Not reported	0% (95% CI: 0%-16.8%)	0 per 1000 (95% CI: 0-8.4 per 1000)

*CI*, Confidence interval; *DBPCFC*, double-blind, placebo-controlled food challenge; *ED*_*05*_, amount of allergen expected to cause objective symptoms in 5% of the population with that allergy; *FC*, food challenge.

These data further support the use of ED to inform the need for PAL. Given that the evidence base is strongest for peanut, with data encompassing over 3000 DBPCFCs reported in the literature (including evidence relating to reproducibility of reaction thresholds and the impact of cofactors), we propose that peanut can be used as an exemplar allergen in terms of hazard characterization at ED_05_ levels of exposure or below. Further work is underway at a global level to consider how reference doses might be used to inform allergen risk management,^110^ and importantly, how this can be communicated in a reassuring way to consumers with food allergy. Whether the nature of symptoms that are experienced at an ED_05_ level of exposure are acceptable to stakeholders, including food-allergic consumers, and could be considered to be a “tolerable risk” requires further consensus.

## Supplementary Data

Online Repository
