# Supplementary material for: Peanut Can Be Used as a Reference Allergen for Hazard Characterization in Food Allergen Risk Management: A Rapid Evidence Assessment and Meta-Analysis
Source: J Allergy Clin Immunol Pract. 2022 Jan;10(1):59–70. doi: 10.1016/j.jaip.2021.08.008 (PMC8790324; doi:10.1016/j.jaip.2021.08.008)
Supplement: Online Repository [file mmc1.docx]

**ROSTRUM:**

**Peanut can be used as a reference allergen for hazard characterization in food allergen risk management: A rapid evidence assessment and meta-analysis**

Paul J Turner FRCPCH PhD, Nandinee Patel MD, Barbara K. Ballmer-Weber MD, Joe L. Baumert PhD, W. Marty Blom PhD, Simon Brooke-Taylor PhD, Helen Brough MD PhD, Dianne E Campbell MD PhD, Hongbing Chen PhD, R. Sharon Chinthrajah MD, René WR Crevel PhD, Anthony E J Dubois MD PhD, Motohiro Ebisawa MD, Arnon Elizur MD, Jennifer D. Gerdts BComm, M. Hazel Gowland PhD,

Geert F. Houben PhD, Jonathan OB Hourihane DM FRCPI, André C. Knulst MD PhD,

Sébastien La Vieille MD MSc, María Cristina López PhD, E.N. Clare Mills PhD, Gustavo A. Polenta PhD, Natasha Purington MS, Maria Said RN, Hugh A. Sampson MD, Sabine Schnadt Dipl.Oec.troph,

Eva Södergren PhD, Stephen L. Taylor PhD, Benjamin C. Remington PhD

**ONLINE SUPPLEMENT**

**METHODOLOGY**

Rapid evidence assessments (REA) are similar to formal systematic reviews, using a structured and rigorous search to evaluate available evidence on a research question. However, they are not as exhaustive as a systematic review, which facilitates a more rapid timeframe. This is achieved through concessions in terms of the range of evidence assessed (e.g. exclusion of unpublished studies), the comprehensiveness of the search, and typically do not include a formal risk of bias assessment.^25^

Remington et al recently demonstrated that open food challenge data (otherwise conducted to PRACTALL consensus criteria) can be used to supplement DBPCFC data for the generation of food-allergic population threshold dose distributions.^E1^ We therefore undertook a REA of the literature to identify studies which have undertaken double-blind, placebo-controlled food challenges (DBPCFC) or open oral food challenges (FC) in allergic individuals (adults and children), conducted in a manner consistent with international consensus criteria. Where necessary, authors were contacted and asked to provide aggregate data or clarifications allowing the inclusion of data for meta-analysis.

**Search strategy**

We searched Medline for articles published between January 1988 and June 2021 which described DBPCFC or open FC to a relevant allergen, using the search terms “double-blind”, “allergy”, “challenge” and the relevant priority allergen: tree nuts, cashew, hazelnut, walnut, cow’s milk, egg, wheat, sesame, soy, soya. There was no registered protocol for this review, but the methods and analyses were planned *a priori*. No language restrictions were made, and we planned to include non-English papers if they met our inclusion criteria. We also reviewed reference lists of included studies and review articles to identify other relevant studies.

**Study selection**

Inclusion criteria were as follows: (i) Participants: children and adults with food allergy to the relevant “priority” allergen; (ii) Intervention: DBPCFC or open FC, conducted in a manner consistent with PRACTALL consensus criteria, to the relevant allergen for diagnostic purposes, with a minimum of ≥10 subjects with objective symptoms at FC and a starting dose at or lower than the reported upper 95% confidence interval for ED_05_; (iii) Outcomes: study-defined cumulative eliciting dose (either maximum tolerated dose or reaction threshold dose, consistent with PRACTALL consensus criteria) or lowest-observed adverse effect level (LOAEL); and the occurrence of anaphylaxis. Studies needed to satisfy all 3 inclusion criteria to be included. Where more than one data series included the same individuals during an overlapping time period, we included the data series with the largest number of individuals where we could be certain that no duplication was present. Excluded studies are reported in Table E11, together with the reason for exclusion.

**Data extraction and analyses**

Authors were contacted where needed to confirm data extracted. Analyses were planned prospectively. For each study, we extracted the number of participants experiencing objective symptoms and/or meeting study-defined challenge stopping criteria to a cumulative dose equal or less than the reported upper 95% confidence interval for ED_05_ for the relevant allergen, and the proportion of those with anaphylaxis (as defined by the study authors). As is usual for safety evaluations, we used a higher (more conservative) mg protein amount (as estimated by the upper 95% confidence interval of the ED_05_) rather than the ED_05_ estimate itself.

We have previously reported that at least for peanut, using different definitions for anaphylaxis (NIAID vs WAO vs presence of significant cardiovascular or lower respiratory symptoms) do not impact on the estimated rate of anaphylaxis at ED05 levels of allergen exposure.^24^ Where individual patient symptom data was available, anaphylaxis was determined according to the WAO 2020 consensus criteria by two independent investigators (PJT, NP). Where needed, a conservative approach was applied, where reactions which could be consistent with WAO criteria were assigned as anaphylaxis, even if study authors did not categorize them as such.

Data were extracted in duplicate; any discrepancies identified between extracted data and published data were resolved by discussion and/or by contacting authors for clarifications. Since this was a rapid evidence assessment, we did not evaluate risk of bias.^25^ Rates were pooled across studies using a generalized linear mixed model in R (metaprop function, metafor package, logit transformation with a random intercept logistic regression model for the summary estimate, with a continuity correction of 0.5). This approach avoids many of the issues surrounding the use of transformations when undertaking meta-analyses of proportions.^E2,E3^ Heterogeneity was quantified using the I^2^ statistic. We conducted meta-analysis even if significant heterogeneity was seen between study estimates, as is the norm when conducting meta-analysis of proportions. The statistical program used for meta-analysis was R, version 4.0.3 (R Project). Binomial confidence intervals were calculated using the Clopper–Pearson interval. Statistical significance was set at 2-sided P < .05.

**Ethical approval**

Ethical approval was not required as this was a post-hoc analysis of anonymized participant data from multiple clinical trials, all of which had their own individual ethics approval.

**REFERENCES**

E1. Remington BC, Westerhout J, Dubois AEJ, Blom WM, Kruizinga AG, Taylor SL, et al. Suitability of low-dose, open food challenge data to supplement double-blind, placebo-controlled data in generation of food allergen threshold dose distributions. Clin Exp Allergy. 2021 Jan;51(1):151-154. doi: 10.1111/cea.13753.

E2. Lin L, Xu C. Arcsine-based transformations for meta-analysis of proportions: pros, cons, and alternatives. Health Science Reports 2020;3:e178.

E3. Schwarzer G, Chemaitelly H, Abu-Raddad LJ, Rücker G. Seriously misleading results using inverse of Freeman-Tukey double arcsine transformation in meta-analysis of single proportions. Res Synth Methods. 2019 Sep;10(3):476-483.

**Table E1**: Studies reporting food challenges to cashew

| CASHEW  Study | Study cohort  (positive FC only) | Anaphylaxis at food challenge | Eliciting dose for patients with anaphylaxis | Anaphylaxis rate in those with objective symptoms to ≤ED_05_ |
| --- | --- | --- | --- | --- |
| McWilliam 2020^32^  Australia  Open FC | 167 children  Mean age 7-8 y | 9 (5%) with lower resp symptoms and treated with epinephrine | Lowest ED 55mg | 0%  (0/9) |
| Saba 2020^33^  France  142 Open FC,  and 5 DBPCFC | 147 children  Median age 8.4 y  IQR 6-12 y  Median ED 39mg | 4 (3%) with lower respiratory symptoms | 3 individuals with ED ≤11.7mg | 8%  (3/39) |
| Brough 2020^29^  Spain, Switzerland, UK  Open FC | 36 children  Median age 5.5 y  IQR 3-10 y | No anaphylaxis | No anaphylaxis | 0%  (0/10)^§^ |
| Sato 2019^34^  Japan  Open FC | 26 young people  Median age 7 y  (range 2-20y)  Median ED 230mg | 4 (15%) with anaphylaxis | 72mg | 0%  (0/2)^§^ |
| Purington 2018^30^  USA  DBPCFC | n=150, children+adults  Median age 8 y  (IQR 6-11)  Median ED 25mg | 9 (6%) with lower resp symptoms (wheezing) | 7.7mg | 5%  (1/20) |
| Elizur 2018^31^  Israel  Open FC | 36 young people, 35 with objective symptoms  Median age 9 y | 8 (22%) with lower resp symptoms | 1mg | 9%  (1/11) |
| Lange 2017^35^  Germany  Open FC | 14 children  Median age 4 y | 3 with CVS or lower respiratory symptoms | No anaphylaxis to <10mg protein | 0%  (0/4) |
| van der Valk 2016^36^  Netherlands  DBPCFC | 137 children  Median 9 y  Median ED 144mg | 7 (5%) with lower respiratory symptoms | 3mg | 6%  (1/16) |
| Blom 2013^27^  Netherlands  DBPCFC | 31 children  Median age 8.3 y  Median ED 203mg | 1 (3%) with lower respiratory symptoms | 760 mg | 0%  (0/11) |

All doses are mg cashew protein. DBPCFC, double-blind, placebo-controlled food challenge; ED, eliciting dose; FC, food challenge. ED_05_ set as upper limit of 95% CI as reported by Houben et al = 9.4mg protein.^6^

^§^Data provided for ≤20mg cashew protein, equivalent to upper 95%CI of ED07 for cashew.^6^

**Table E2:** Studies reporting food challenges to hazelnut

| HAZELNUT  Study | Study cohort  (positive FC only) | Anaphylaxis at food challenge | Eliciting dose for patients with anaphylaxis | Anaphylaxis rate in those with objective symptoms to ≤ED_05_ |
| --- | --- | --- | --- | --- |
| Brough 2020^29^  Spain, Switzerland, UK  Open FC | 30 children  Median age 5.5 y  (IQR 3-10 y) | No anaphylaxis | No anaphylaxis | 0%  (0/6)^§^ |
| Moraly 2020^37^  France  DBPCFC | 100 children  Median age 5 y  Median ED 106 mg | 1 (1%) | ~204mg | 0%  (0/13) |
| Purington 2018^30^  USA  DBPCFC | n=65, children+adults  33 with obj symptoms  Median age 8 y  (IQR 7-11 y)  ED 25 mg | No anaphylaxis | No anaphylaxis | 0%  (0/26) |
| Masthoff 2018^38^  Netherlands  DBPCFC | 108 children & adults  Median age 14 y  Median ED 662 mg | Not reported | Not reported | Not reported |
| Elizur 2018^31^  Israel  Open FC | 13 young people, 12 with objective symptoms  Median age 9 y | 2 (15%) with lower respiratory symptoms | 20mg, 3840 mg | 20%  (1/5) |
| Ballmer-Weber 2015^28,39^  Europe  DBPCFC | 87 adults & children  Median age 31 y  Median ED 1433 mg | 10 (11%)  study-reported anaphylaxis | 0.333 mg | 9%  (1/11) |
| Blom 2013^27^  Netherlands  DBPCFC | 23 children  Median age 6.5 y  Median ED 186mg | No anaphylaxis | No anaphylaxis | 0%  (0/15) |
| Flinterman 2006^40^  Netherlands  DBPCFC | 12, 8 with objective symptoms  Median age 6 y  Median ED >4411mg | 1 (13%) with lower respiratory symptoms | >4.4g protein | 0%  (0/0) |

All doses are mg hazelnut protein. DBPCFC, double-blind, placebo-controlled food challenge; ED, eliciting dose; FC, food challenge. ED_05_ set as upper limit of 95% CI as reported by Houben et al = 16mg protein.^6^

^§^Reference used in these studies was a cumulative dose of 30mg protein, which is equivalent to upper 95%CI of ED_07_ for hazelnut.^6^

**Table E3**: Studies reporting food challenges to walnut

| WALNUT  Study | Study cohort  (positive FC only) | Anaphylaxis at food challenge | Eliciting dose for patients with anaphylaxis | Anaphylaxis rate in those with objective symptoms to ≤ED_05_ |
| --- | --- | --- | --- | --- |
| Goldberg 2021^41^  Israel  Open FC | 120 young people, 106 with objective symptoms  Median age 8  (range, 4-27 years)  Median ED 205mg | 12 (11%) with lower respiratory symptoms | Lowest ED 20mg | 4%  (1/23)  (cumulative dose 25mg) |
| Brough 2020^29^  UK, Spain, Switzerland  Open FC | 36 children  Median age 5.5 y  (IQR 3-10 y) | 1/36 (3%) with stridor | 30mg | 14%  (1/7)^§^ |
| Remington 2020^42^  Netherlands  DBPCFC | 41 children  Median age 9 y  Median ED 78mg | 1 (2%) with lower resp symptoms | 225mg | 0%  (0/8) |
| Purington 2018^30^  USA  DBPCFC | n=120, children+adults  41 with obj symptoms  Median age 8 y  (IQR 6-11 y)  Median ED 25 mg | 3 (3%) with lower resp symptoms (wheezing) | 0.1mg, 1.7mg | 6%  (2/35) |
| Blankestijn 2017^43^  Netherlands  DBPCFC | 33 adults  Median age 30 y  (IQR 25-35 y)  Median ED 361mg | 7 (21%) with laryngeal or lower resp symptoms | 33.6mg | 0%  0/2 |

All doses are mg walnut protein. ED, eliciting dose; FC, food challenge.

ED_05_ set as upper limit of 95% CI as reported by Houben et al = 13mg protein.^6^

^§^First dose of used in these studies was ≈30mg protein, which is equivalent to upper 95%CI of ED_08_ for walnut.^6^

**Table E4**: Studies reporting food challenges to sesame

| SESAME  Study | Study cohort  (positive FC only) | Anaphylaxis at food challenge | Eliciting dose for patients with anaphylaxis | Anaphylaxis rate in those with objective symptoms to ≤ED_05_ |
| --- | --- | --- | --- | --- |
| Saf 2020^44^  USA  Open FC | 106 children  (ED not available in 6)  Median ED 500mg | 23 (22%) NIAID  13 (12%) lower resp symptoms | Lower respiratory symptoms: 191mg  NIAID: 3mg  (abdo pain+urticaria) | 0%  (0/10)  [lower resp symptoms] |
| Salari 2020^45^  Iran  Open FC | 16 adults  Median age 38 y  Median ED 133mg | 3 x Ring III  1 x Ring IV | No anaphylaxis to <133mg | 0%  (0/5) |
| Brough 2020^29^  UK, Spain, Switzerland  Open FC | 8 children  Median age 5.5 y  (IQR 3-10 y) | No anaphylaxis | No anaphylaxis | 0%  (0/4) |
| Yanagida 2019^46^  Japan  Open FC | 18 children  Median age 6.1 y  Median ED 609mg | 6/18 (33%) | No patient reacted to <152mg | 0%  (0/0) |
| Appel 2018^47^  Israel  Open FC | 40 children, median 6.8y  12 adults, median 19 y  Median ED 180mg | 12 (23%) NIAID  3 (6%) lower resp symptoms | 3mg, 240mg, 480mg | 5.6%  (1/18) |
| Purington 2018^30^  USA  DBPCFC | n=30, children+adults  Median ED 25mg | 1 (3%) lower resp | 500mg | 0%  (0/17) |
| Chan 2017^48^  Australia  Open FC | 20 children, age 4 y | No anaphylaxis | No anaphylaxis | 0%  (0/5) |
| Dano 2015^49^  France  DBPCFC | 5 children + 9 adults  Median ED 85mg | 2 patients with “wheezing” | 2.6mg (had “minimal wheezing);  340mg in 2^nd^ patient. | 25%  (1/4) |
| Leduc 2006^50^  France  DBPCFC | 5 children + 10 adults  Median ED 164mg | 4 lower resp (27%) | Lowest ED in patients with anaphylaxis = 164mg | 0%  (0/3) |

All doses are mg sesame protein. ED, eliciting dose; FC, food challenge.

ED_05_ set as upper limit of 95% CI as reported by Houben et al = 58mg protein.^6^

**Table E5**: Studies reporting FC to cow’s milk

| COW’S MILK  Study | Study cohort  (positive FC only) | Anaphylaxis at food challenge | Eliciting dose for patients with anaphylaxis | Anaphylaxis rate in those with objective symptoms to ≤ED_05_ |
| --- | --- | --- | --- | --- |
| Turner 2021^51^  UK, Spain, Ireland  DBPCFC | 119 children  Median age 1.0 y  (range 0.2–16 y) | 15/119 (13%) anaphylaxis | 0.5mg (2 patients) | 13%  (2/15) |
| Inuo 2019^52^  Japan  DBPCFC | 25 children  Median age 4.3y  Median ED 61mg | 12 (48%) with lower resp symptoms | 12.2mg | 0%  (0/1) |
| Purington 2018^30^  USA  DBPCFC | n=67, children+adults  data available for 51  Median ED 327mg | 5 (10%) with lower resp symptoms | 33mg | 0%  (0/19) |
| Yanagida 2017^53^  Japan  DBPCFC | 164 children  Median 8.6 y  Median ED 840mg | 89 (54%) with Sampson IV/V reactions | 5.1mg | 13%  (1/8) |
| Blom 2013^27^  Netherlands  DBPCFC | 87 children  Median 2.4y  Median ED 389mg | 9 (12%) with lower resp symptoms | Lowest ED 1.75mg (not treated with epinephrine), next lowest ED 89mg | 14%  (1/7) |
| Dambacher 2013^54^  Netherlands  DBPCFC | 21 children  Median age 0.7 y  Median ED 1080mg | 1 (5%) with lower resp symptoms | 1620mg | N/A  1^st^ FC dose ≈18mg |
| Lee et al, 2013^55^  South Korea  DBPCFC | 31 children  Mean age 0.7 y  Median ED 1080mg | 3 (10%) anaphylaxis | No reactions to ≤33mg | 0%  (0/0) |
| EuroPrevall 2013^42,56^  Europe (16 countries)  DBPCFC | 67 children, 2 adults  Median age 1.2 y  Median ED 123mg | 6 (8.7%)  anaphylaxis | Lowest ED for anaphylaxis 33.33mg | 0%  (0/12) |
| Keet 2012^57^  USA  DBPCFC | 30 children  Median age 8 y  Median ED 51mg | 4 (13%) treated with epinephrine | Lowest ED treated with epinephrine  = 11.1mg. | 0/5 to ≤1.1mg  1/14 to ≤11.1mg |
| Rolinck-Werninghaus 2012^58^  Germany  DBPCFC and open FC | 305 children  Median age 1.0 y  Median ED 1443mg | 19 (6.2%) with Sampson IV reactions | 1 Grade IV reaction to 3mg, 1 to 13mg, 2 to 43mg. | 3.4%  (1/29) |
| Pajno 2010^59^  Italy  DBPCFC | 30 children  Median age 10 y  Median ED 100mg | No anaphylaxis | No anaphylaxis | 0%  (0/3) |
| Caminiti 2009^60^  Italy  DBPCFC | 11 children  Mean age ~8 y  Median ED 145mg | 2 (18%) with lower resp symptoms | No anaphylaxis to ≤13mg | 0%  (0/0) |
| Lam 2008^61^  Netherlands  DBPCFC | 10 adults  Median age 40 y | 3 (30%) with lower resp symptoms | No anaphylaxis to ≤30mg | 0%  (0/1) |
| Morisset 2007^62^  France  Single blinded FC | 12 children, 1-6.5 y  - all tolerated 2g CMP 6m prior to FC | No child had lower resp symptoms | No anaphylaxis to ≤900mg | 0%  (0/0) |
| Patriarca 2007^63^  Italy  DBPCFC | 11 children  Median age 6 y  Median ED 102mg | 3 (27%) with lower resp symptoms | Lowest ED for anaphylaxis 34mg | 0%  (0/1) |
| Morisset 2003^64^  France  DBPCFC | 52 children and 7 adults  Age not specified. | 6 (10%) lower resp symptoms, 1 with fall in BP | No anaphylaxis to ≤9.6mg | 0%  (0/1) |
| Baehler 1996^65^  Canada  DBPCFC | 10 children  Mean age 3 y  Median ED 324mg | 3 (30%) with lower resp symptoms | 180mg | 0%  (0/0) |

All doses are mg cow’s milk protein. ED, eliciting dose; FC, food challenge.

ED_05_ set as upper limit of 95% CI as reported by Houben et al = 6.6mg protein.^6^

**Table E6**: Studies reporting FC to hen’s egg

| HEN’s EGG  Study | Study cohort  (positive FC only) | Anaphylaxis at food challenge | Eliciting dose for patients with anaphylaxis | Anaphylaxis rate in those with objective symptoms to ≤ED_05_ |
| --- | --- | --- | --- | --- |
| Kim 2020^69^  USA  DBPCFC  *EW powder* | 51 children allergic to baked egg at FC | No anaphylaxis | No anaphylaxis | 0%  (0/2) |
| Takaoka 2019^70^  Japan  Open FC  *Hard boiled egg* | 33 children  Median age 6 y Median ED 130mg | 1 (3%) with lower resp symptoms | 130mg | 0%  (0/0) |
| Purington 2018^30^  USA  DBPCFC | n=63, children+adults  Median ED 8mg | 7 (22%) with CVS or lower resp symptoms | No anaphylaxis to ≤50mg | 0%  (0/10) |
| Yanagida 2017^53^  Japan  DBPCFC | 152 children  Median 8.6 y  Median ED 3100mg | 46 (30%) with Sampson Grade IV/V reactions | No anaphylaxis to ≤194mg | 0%  (0/0) |
| Chan 2017^71^  Australia  DBPCFC  *Raw egg* | 57 children,  age ~4 years | No anaphylaxis | No anaphylaxis | N/A  1^st^ FC dose ≈130mg |
| Bellach 2017^72^  Germany  DBPCFC  *Pasteurized EW powder* | 16 children,  aged 4-6 months  Median ED 516mg | 4 (25%) anaphylaxis | Lowest ED 52mg | 0%  (0/1) |
| Pérez-Rangel 2017^73^  Spain  DBPCFC  *Dehydrated EW* | 33 children  Mean age 10.4 y  Median ED 311mg | 4 had lower resp symptoms | No anaphylaxis ≤19mg | 0%  (0/0) |
| Xepapadaki 2016^74^  Europe  DBPCFC  *Egg white powder* | 84 children Mean age 10.5 m  Median ED 333mg | 9/78 with anaphylaxis | 30mg | 0%  (0/12) |
| Ballmer-Weber 2016^75^  Italy/Germany/Switzerland  DBPCFC  *EW powder* | 24 children  Median age 3.2 y  Median ED 79mg | 3/24 (12.5%) | No anaphylaxis to ≤2000mg | 0%  (0/3) |
| Vazquez-Ortiz 2014^76^  Spain  DBPCFC  *Boiled EW* | 82 children  30 controls  52 active therapy | Anaphylaxis in:  18/30 controls  30/50 active | In control group, no anaphylaxis to ≤120mg | N/A  1^st^ FC dose ≈120mg |
| Turner 2013^77^  Australia  Open FC  *Baked egg in muffin* | 86 children  Median age 4.8y  Median ED 390mg | 12 (14%) anaphylaxis | Lowest ED ≈ 75mg.  No anaphylaxis to 1^st^ dose of 40mg | N/A  1^st^ FC dose ≈40mg |
| Dello Iacono 2013^78^  Italy  DBPCFC  *Raw egg* | 13 children  Median age 1.8 y  Median ED 13mg | 4/10 in control group had anaphylaxis | No anaphylaxis to ≤5mg | 0%  (0/0) |
| Meglio 2013^79^  Italy  DBPCFC  *Raw egg* | 16 children  Median age 7.1 y  Median ED 15.4mg | 3 (19%) with lower resp symptoms | ~20mg | 0%  (0/1) |
| EuroPrevall 2013^80^  Europe  DBPCFC  *EW powder* | 21 patients >3.5 y  13 children, 8 adults  (median age 12 y) | Not stated | Lowest ED 33.3mg  No anaphylaxis to ≤3.33mg | 0%  (0/2) |
| Blom 2013^27^  Netherlands  DBPCFC  *Whole egg powder* | 53 children  Median age 4.5y  Median ED 425mg | 1 (2%) with lower resp symptoms | 2118mg | 0%  (0/6) |
| Rolinck-Werninghaus 2012^58^  Germany  DBPCFC and open FC  *Pasteurized EW powder* | 313 children  Median age 1.6 y  Median ED 205mg | 21 (7%) with Sampson Grade IV reactions | 4 had Grade IV reactions to 5mg, 1 to 19mg, 4 to 65mg. | 14%  (4/28) |
| Clark 2011^81^  UK  Open FC  *Uncooked whole egg* | 61 children  Median age 5.5y | 9 (15%) lower resp | No anaphylaxis to ≤133mg | N/A  1^st^ FC dose ≈60mg |
| Benhamou 2008^82^  Switzerland  Mix of open and DBPCFC  *Raw egg /*  *boiled egg* | 35 children  Median age 4y  26 FC to *raw egg*  9 FC to *boiled egg* | Sampson grade III/IV/V in:  - 10/26 *raw egg*  - 3/9 *boiled egg* | Only 1 patient reacted to ≤65mg, with Grade III/IV/V reaction | N/A  1^st^ FC dose ≈60mg |
| Patriarca 2007^63^  Italy  DBPCFC  *EW* | 10 children with objective symptoms  Median age 9 y  Median ED 21.5mg | No anaphylaxis | No anaphylaxis | 0%  (0/3) |
| Morisset 2007^62^  France  Single Blinded FC  *Raw EW* | 15 children  Mean age 3.5±1.7y  All tolerated ~100mg egg protein 6m prior to FC | 3 (20%) with lower resp symptoms | No anaphylaxis to <300mg | N/A  1^st^ FC dose ≈66mg |
| Caffarelli 1995^83^  Italy  DBPCFC  *Dried egg powder* | 13 children  Median age 1.8 y  Median ED 6g | 2 anaphylaxis | Both occurred to >9mg | 0%  (0/3) |

All doses are mg egg protein unless otherwise stated. ED, cumulative eliciting dose; EW, Egg white; FC, food challenge. ED_05_ set as upper limit of 95% CI as reported by Houben et al = 5.3mg protein.^6^

**Table E7**: Studies reporting FC to wheat.

| WHEAT  Study | Study cohort  (positive FC only) | Anaphylaxis at FC | Eliciting dose for patients with anaphylaxis | Anaphylaxis rate in those with objective symptoms to ≤ED_05_ |
| --- | --- | --- | --- | --- |
| Nowak-Węgrzyn 2019^88^  USA  DBPCFC | n=47, children+adults  Median age 8.7 y  Median ED 143mg | 26 (55%) treated with epinephrine | Not reported | 0%  (0/11)^§^ |
| Purington 2018^30^  USA  DBPCFC | n=13, children+adults  Median ED 33mg | 3 (23%) with lower resp symptoms | No anaphylaxis ≤25mg | 0%  (0/5) |
| Yanagida 2017^53^  Japan  DBPCFC | 50 children  Median age 8.1 y  Median ED 159mg | 35 (70%) Sampson Grade IV/V reaction | 52mg  No anaphylaxis to ≤40mg | 0%  (0/1) |
| Okada 2016^89^  Japan  Open FC | 36 children with reaction thresholds ≤53mg wheat protein.  Median age 2.9 y | 13 (72%) with persistent cough | 6/36 reacted to 13.3mg with persistent cough | 86%  (6/7) |
| Nilsson et al, 2015^90^  Sweden  Open FC | 26 children  Median age 5.0 y  Median ED not stated | 10 (38%) with resp symptoms | No anaphylaxis to ED<50mg | 0%  (0/0) |
| Christensen 2014^91^  Denmark  Both open and DBPCFC | 15 children, median 2.5y  6 adults, median 40 y  Median ED 1600mg | 2 (10%) with lower resp symptoms | 400mg, 600mg | 0%  (0/1) |
| Cianferoni 2013^87^  USA  Open FC | 39 children  Mean age 3.3±1.7 y  Median ED >1000mg | 17 (44%) NIAID anaphylaxis | 97mg  No anaphylaxis to ≤25mg | 0%  (0/0) |
| Rolinck-Werninghaus 2012^58^  Germany  DBPCFC and open FC | 88 children  Median age 1.4 y  Median ED 4.03g | 9 (10%) with Sampson IV reaction | 2 had anaphylaxis to 39mg, remainder to >100mg. | 0%  (0/5) |
| Ito 2008^92^  Japan  Open FC | 21 children  Median age 1.4 y  Median ED 208mg | 8 (38%) with lower resp symptoms | 3 patients had EDs <100mg:  2.6, 26, 78mg | 67%  (2/3) |
| Scibilia 2006^93^  Denmark, Italy  DBPCFC | 13 adults  Median age not stated  Median ED 390mg | 3 (23%) with lower resp symptoms | 60mg | 0%  (0/2) |

All doses are mg wheat protein. ED, eliciting dose; FC, food challenge. ED05 set as upper limit of 95% CI as reported by Houben et al = 25mg protein.^6^ ^§^Data relates to initial escalation day where cumulative dose = 17mg protein, n=44

**Table E8**: Studies reporting FC to fish

| FISH  Study | Study cohort  (positive FC only) | Anaphylaxis at FC | Eliciting dose for patients with anaphylaxis | Anaphylaxis rate in those with objective symptoms to ≤ED_05_ |
| --- | --- | --- | --- | --- |
| Sørensen 2017^96^  Norway   - *cod* - *salmon* - *mackerel*   DBPCFC | 34 children  Mean age 11.6±3.0 y  32 *cod* (median ED 1g)  23 *salmon* (median ED 1.5g)  19 *mackerel* (median ED 2g) | 12/32 (38%)  5/23 (22%)  7/19 (37%) | Lowest EDs for anaphylaxis:  3 @ 133mg  2 @ 1000mg  1 @ 133mg | *Cod*: 0/2 @ 13mg  3/12 @ ≤133mg  *Salmon*: 0/2 @ 13mg  0/7 @ ≤133mg  *Mackerel* 0/1 @ 13mg  1/2 @ ≤133mg  Overall:  0% (0/5) for 13mg |
| Ballmer-Weber 2015^28^  Iceland, Greece, France, Spain, Lithuania   - *cod*   DBPCFC | 16 children (median age 2.5y) and 5 adults (median age 24y) with objective symptoms  Median ED 1.7g | 3/23 (17%) | EDs reported:  33mg  1219mg  1433mg | 20% (1/4) for ≤133mg |
| Helbling 1999^97^  Switzerland   - *cod* - *snapper* - *catfish*   DBPCFC | 9 adults, mean age 31 y  5 *cod* (median ED 720mg)  4 *snapper* (median ED 550mg)  6 *catfish* (median ED 1.8g) | 1/5 (20%)  1/4 (24%)  1/6 (17%) | EDs reported:  720mg  880mg  180mg | For catfish: 0% (0/1)  Lowest dose for cod/snapper ≈180mg |

All doses are mg protein unless otherwise stated. ED_05_ set as upper limit of 95% CI as reported by Houben et al = 102mg protein, discrete ED_05_ = 12mg protein.^6^

**Table E9**: Studies reporting FC to prawn/shrimp

| SHELLFISH  Study | Study cohort  (positive FC only) | Anaphylaxis at FC | Eliciting dose for patients with anaphylaxis | Anaphylaxis rate in those with objective symptoms to ≤ED_05_ |
| --- | --- | --- | --- | --- |
| Thalayasingam 2015^98^  Singapore   - *glass prawn* - *tiger prawn*   Open FC | 25 adults  Median age 30 y  Median ED 1.2g  Median ED 1.02g | 4/25 (16%)  2/17 (12%) | Lowest EDs reported:  24mg, 720mg, 1.2g (x2)  609mg (x2) | 25% (6/24) for <1.2g  12.5% (1/8) for <0.5g |
| Ballmer-Weber 2015^28^  Iceland, France, Spain, Switzerland  *N. Atlantic prawn*  DBPCFC | 2 children (both age 10y) and 10 adults (median age 29y) with obj. symptoms  Median ED 10.1g | No anaphylaxis | N/A | 0% (0/0)  No objective reactions below 4g |
| Daul 1988^99^  USA  *Shrimp*  Open FC | 21 adults  Mean ED 10.7g | 4 lower resp symptoms | Nil <4g | 0%  (0/0)  No objective reactions below 4g |

All doses are mg protein. ED_05_ set as upper limit of 95% CI as reported by Houben et al = 1850mg protein for shrimp, discrete ED_05_ = 280mg protein.^6^

**Table E10**: Studies reporting FC to soybean

| SOYBEAN  Study | Study cohort  (positive FC only) | Anaphylaxis at FC | Eliciting dose for patients with anaphylaxis | Anaphylaxis rate in those with objective symptoms to ≤ED_05_ |
| --- | --- | --- | --- | --- |
| Treudler 2016^101^  Germany, Switzerland  DBPCFC | 82 adults, 58 with objective symptoms  Mean age 37±14 y  Median ED 4.7g | 3 with objective fall in peak flow, 3 with drop of blood pressure >20 mmHg | No anaphylaxis to ≤76mg | 0%  (0/10) |
| Blom 2013^27^  Netherlands  DBPCFC | 10 children, 3 with objective symptoms  Median age 10y | No anaphylaxis | No anaphylaxis | 0%  (0/1) |
| Rolinck-Werninghaus 2012^58^  Germany  DBPCFC and open FC | 51 children  Median age 1.1 y  (range 0.3–16 y)  Median ED 5.2g | 2 (4%) with Sampson Grade IV reactions | 2 anaphylaxis to 5200mg | 0%  (0/5) |
| Ballmer-Weber 2007^102^  Switzerland, Denmark, Italy  DBPCFC | 23 adults, 11 with objective symptoms  Mean age 37±14 y  Median ED 4.6g | 3 with drop in peak flow or blood pressure >20 mmHg | Lowest ED 867mg with lower resp symptoms | 0%  (0/4) |
| Zeiger 1999^103^  USA  DBPCFC | 10 children  Median age 1.7 y | 2 (20%) with lower resp symptoms | Lowest ED 990mg with lower resp symptoms | 0%  (0/0) |

All doses are mg soya protein. ED_05_ set as upper limit of 95% CI as reported by Houben et al = 76mg protein.^6^

**Table E11**: Excluded studies which otherwise met the study inclusion criteria

| **Allergen** | **Study** | **Reason for exclusion** |
| --- | --- | --- |
| Sesame | Goldberg 2021. PAI  doi: 10.1111/pai.13533 | Overlap with Appel et al 2018 |
| Egg | Palosuo 2021 JACI Pract. 9(5):1892-1901.e1 doi: 10.1016/j.jaip.2021.01.020 | Overlap with Palosuo et al 2018 |
| Cashew, hazelnut, walnut, sesame | Santos 2021. JACI Pract. 9(5):2016-27.e6  doi: 10.1016/j.jaip.2020.12.039 | Overlap with Brough 2020 |
| Sesame | Sokol 2020 PAI 31(2):214-218  doi: 10.1111/pai.13143 | Reports only 3 FC. |
| Cashew, hazelnut, walnut | Duan 2020. Allergy 76(6):1800-1812  doi: 10.1111/all.14695 | FC protocol started at 3mg but 2^nd^ dose ≥100mg. |
| Walnut | Elizur 2020. JACI Pract. 8:157-165.e2  doi: 10.1016/j.jaip.2019.08.038 | Included in Goldberg et al 2021 |
| Egg | Grimshaw 2020. JACI Pract. 8(4):1341-8  doi: 10.1016/j.jaip.2019.11.040 | Included in Xepapadaki et al, 2016. |
| Walnut | Ballmer-Weber 2019. JACI Pract. 7: 1560-7  doi: 10.1016/j.jaip.2019.01.029 | Required data not in paper and no longer accessible. |
| Walnut | Elizur 2019. Lancet Child Adolesc Health 3: 312-21. doi: 10.1016/S2352-4642(19)30029-X | Included in Goldberg et al 2021 |
| Cow’s milk | Ruinemans-Koerts 2019. Clin Exp Allergy 49(3):350-6. doi: 10.1111/cea.13307 | Required data not in paper and no response from authors |
| Sesame | Nachshon 2019. JACI Pract. 8(8):2775-81  doi: 10.1016/j.jaip.2019.05.031 | Overlap with Appel et al 2018 |
| Cow’s milk, egg, peanut, cashew, hazelnut | Pettersson 2018. Allergy 73:1532-40  doi: 10.1111/all.13423 | Unable to clarify overlap with other datasets  e.g. Blom et al 2013. |
| Egg | Palosuo 2018 29(6):637-643  doi: 10.1111/pai.12954 | Data not provided in time for inclusion |
| Hazelnut | Datema 2018. Allergy 73(3):549-559  doi: 10.1111/all.13328 | Included in Ballmer-Weber et al, 2015. |
| Cashew | Inoue 2018. Int Arch Allergy Immunol. 175(1-2):99-106. doi: 10.1159/000486120 | Overlap with Sato et al 2019 |
| Cow’s milk | ELBadawy 2017  Egypt J Immunol. 24(2):109-125 | Required data not in paper and no response from authors |
| Cow’s milk | Ebrahimi 2017. Iran J Allergy Asthma Immunol. 16(3):183-192 | Required data not available: no conversion factor to mg protein |
| Cow’s milk, egg, peanut, cashew, hazelnut, walnut | Andorf 2017. JACI Pract 5:1325-34  doi: 10.1016/j.jaip.2017.01.016 | Required data not in paper and no response from authors |
| Sesame | Li 2017. Ann Allergy Asthma Immunol. 119:285-7. doi: 10.1016/j.anai.2017.06.013 | Less than 10 FC for any given food matrix. |
| Hazelnut | Eller 2016. Allergy 71:556–62  doi: 10.1111/all.12820 | Included in Eller 2012 |
| Hazelnut | Buyuktiryaki 2016. JACI Pract. 4:265-72  doi: 10.1016/j.jaip.2015.12.012 | Required data not in paper and no response from authors |
| Hazelnut | Beyer 2015. Allergy 70(1):90-8  doi: 10.1111/all.12530 | Required data not in paper and no longer available. |
| Egg | Escudero 2015. Clin Exp Allergy 45:1833-43  doi: 10.1111/cea.12604. PMID: 26236997 | Initial dose unclear but >ED_05_ for egg. |
| Egg | Libbers 2013. Clin Exp Allergy 43(9):1067-70 doi: 10.1111/cea.12164. PMID: 23957342 | Data not available for cow’s milk and egg. For sesame, <10 FC for any given food matrix. |
| Hazelnut | Masthoff 2013. JACI 132(2):393-9  doi: 10.1016/j.jaci.2013.02.024 | ?Included in Masthoff et al, 2018 |
| Cow’s milk | Alessandri 2012. PLoS ONE 7(7):e40945  doi: 10.1371/journal.pone.0040945 | Required data not in paper and no longer available. |
| Cow’s milk | Alvaro 2012. Eur J Pediatr. 171(9):1389-95  doi: 10.1007/s00431-012-1739-z | Required data not in paper and no longer available. |
| Hazelnut | Eller 2012. Ann Allergy Asthma Immunol. 108:332-6. doi: 10.1016/j.anai.2012.03.010 | Required data not in paper and no response from authors |
| Egg | Ahrens 2010. Allergy 65(12):1554-7  doi: 10.1111/j.1398-9995.2010.02429.x | Required data not in paper and no response from authors |
| Hazelnut | Worm 2009. Clin Exp Allergy 39:159-66  doi: 10.1111/j.1365-2222.2008.03143.x | Less than 10 participants with +ve FC in any single group |
| Cow’s milk | Longo 2008. JACI 121(2):343-7  doi: 10.1016/j.jaci.2007.10.029 | Required data not in paper and no longer available |
| Wheat | Pastorello 2007. Int Arch Allergy Immunol. 144(1):10-22. doi: 10.1159/000102609 | Overlap with Scibilia et al, 2006 |
| Cow’s milk | Niggemann 2004. J Investig Allergol Clin Immunol. 14(2):98-103. | Required data not in paper and no response from authors |
| Cow’s milk, egg, sesame | Morisset 2003. Clin Exp Allergy. 33:1046-51. doi: 10.1046/j.1365-2222.2003.01734.x | Data not available |
| Cow’s milk | Rancé 2002. Arch Pediatr. 9 S3:402s-407s.  doi: 10.1016/s0929-693x(02)00151-3 | Required data not in paper and no longer available |
| Cow’s milk | Giampietro 2001. PAI 12(2):83-6.  doi: 10.1034/j.1399-3038.2001.012002083.x | Required data not in paper and no response from authors |
| Cow’s milk | Niggemann 2001. PAI 12(2):78-82.  doi: 10.1034/j.1399-3038.2001.012002078.x | Required data not in paper and no response from authors |

**FIGURE LEGENDS (ONLINE SUPPLEMENT)**

**Fig E1**: Meta-analysis of studies reporting the proportion of individuals with **cashew** allergy reacting with objective symptoms to ≤9.4mg cashew protein (the upper limit of 95% CI for the ED_05_ for cashew) with anaphylaxis.

**Fig E2:** Meta-analysis of studies reporting the proportion of individuals with **hazelnut** allergy reacting with objective symptoms to ≤16mg hazelnut protein (the upper limit of 95% CI for the ED_05_ for hazelnut) with anaphylaxis.

**Fig E3**: Meta-analysis of studies reporting the proportion of individuals with **walnut** allergy reacting with objective symptoms to ≤13mg walnut protein (the upper limit of 95% CI for the ED_05_ for walnut) with anaphylaxis.

**Fig E4**: Meta-analysis of studies reporting the proportion of individuals with **sesame** allergy reacting with objective symptoms to ≤58mg sesame protein (the upper limit of 95% CI for the ED_05_ for sesame) with anaphylaxis.

**Fig E5**: Meta-analysis of studies reporting the proportion of individuals with **cow’s milk** allergy reacting with objective symptoms to ≤6.6mg cow’s milk protein (the upper limit of 95% CI for the ED_05_ for cow’s milk) with anaphylaxis.

**Fig E6**: Meta-analysis of studies reporting the proportion of individuals with **hen’s egg** allergy reacting with objective symptoms to ≤5.3mg egg protein (the upper limit of 95% CI for the ED_05_ for hen’s egg) with anaphylaxis.

**Fig E7**: Meta-analysis of studies reporting the proportion of individuals with **wheat** allergy reacting with objective symptoms to ≤25mg wheat protein (the upper limit of 95% CI for the ED_05_ for wheat) with anaphylaxis.
